# A mixture model for signature discovery from sparse mutation data

**DOI:** 10.1186/s13073-021-00988-7

**Published:** 2021-11-01

**Authors:** Itay Sason, Yuexi Chen, Mark D.M. Leiserson, Roded Sharan

**Affiliations:** 1 Blavatnik School of Computer Science, Tel Aviv University, Tel Aviv, 69978 Israel; 2 Department of Computer Science and Center for Bioinformatics and Computational Biology, University of Maryland, College Park, 20742 MD USA

**Keywords:** Mutational signatures, Probabilistic modeling, Gene panel sequencing

## Abstract

Mutational signatures are key to understanding the processes that shape cancer genomes, yet their analysis requires relatively rich whole-genome or whole-exome mutation data. Recently, orders-of-magnitude sparser gene-panel-sequencing data have become increasingly available in the clinic. To deal with such sparse data, we suggest a novel mixture model, Mix. In application to simulated and real gene-panel sequences, Mix is shown to outperform current approaches and yield mutational signatures and patient stratifications that are in higher agreement with the literature. We further demonstrate its utility in several clinical settings, successfully predicting therapy benefit and patient groupings from MSK-IMPACT pan-cancer data. Availability: https://github.com/itaysason/Mix-MMM.

**Supplementary Information:**

The online version contains supplementary material available at (10.1186/s13073-021-00988-7).

## Background

Each cancer genome is shaped by a combination of processes that introduce mutations over time [1, 2]. The incidence and etiology of these mutational processes may provide insights into tumorigenesis and personalized therapy. It is thus important to uncover the characteristic signatures of active mutational processes in patients from their patterns of single base substitutions [3–5]. Some such mutation signatures have been linked to exposure to specific carcinogens, such as tobacco smoke [6] and ultraviolet radiation [3]. Other mutation signatures arise from deficient DNA damage repair pathways. By serving as a proxy for the functional status of the repair pathway, mutational signatures provide an avenue around traditional driver mutation analyses. This is important for personalizing cancer therapies, many of which work by causing DNA damage or inhibiting DNA damage response or repair genes [7–10], because the functional effect of many variants is hard to predict. Indeed, a recent study [11] estimated a > 4-fold increase in the number of breast cancer patients with homologous recombination repair deficiency—making them eligible for PARP inhibitors [12]—when using mutational signatures compared to current approaches. Thus, understanding the signatures of mutational processes may lead to the development of many effective diagnostic and treatment strategies.

Statistical models for discovering and characterizing mutational signatures are crucial for realizing their potential as biomarkers in the clinic. A broad catalog of mutational signatures in cancer genomes was only recently revealed through computational analysis of mutations in thousands of tumors. Alexandrov et al. [3, 4] were the first to use non-negative matrix factorization (NMF) to discover mutation signatures. Subsequent methods have used different forms of NMF [13–16] or focused on inferring the exposures (aka refitting) given the signatures and mutation counts [17–19]. A more recent class of approaches borrows from the world of topic modeling, aiming to provide a probabilistic model of the data so as to maximize the model’s likelihood [20–23].

These previous methods are applicable for whole-genome or even whole-exome sequencing (WGS or WXS). However, they cannot handle very sparse data as obtained routinely in targeted (gene panel) sequencing assays [24]. There is only a single method, SigMA, that attempts to address this challenge [24] by relying on whole-genome training data to interpret sparse samples and predict their homologous recombination deficiency status. However, SigMA still suffers from the fact that not all cancer types have available whole-genome sequencing data.

Here, we present the first model that can handle sparse targeted sequencing data *without pre-training* on rich data. Our model simultaneously clusters the samples and learns the mutational landscape of each cluster, thereby overcoming the sparsity problem. Using synthetic and real targeted sequencing data, we show that our method is superior to current non-sparse approaches in signature discovery, signature refitting and patient stratification. We further demonstrate the utility of our model in several clinical settings.

## Methods

### Preliminaries

We follow previous work and assume that somatic mutations in cancer fall into *M*=96 categories, denoting the mutation identity and its flanking bases [3]. These mutations are assumed to be the result of the activity of *K* (a hyper-parameter) mutational processes, each of which is associated with a signature $S_{i}=(e_{i}(1) \dots e_{i}(M))$ of probabilities to emit each of the mutation categories. Henceforth, we denote the mutation categories observed in a given tumor *n* by $O^{n} = (o_{1}\dots o_{T_{n}})$, and we assume that this sequence was emitted by the (hidden) signature sequence $Z^{n} = (z_{1}\dots z_{T_{n}})$.

### Multinomial mixture model (MMM)

**Fig. 1:**
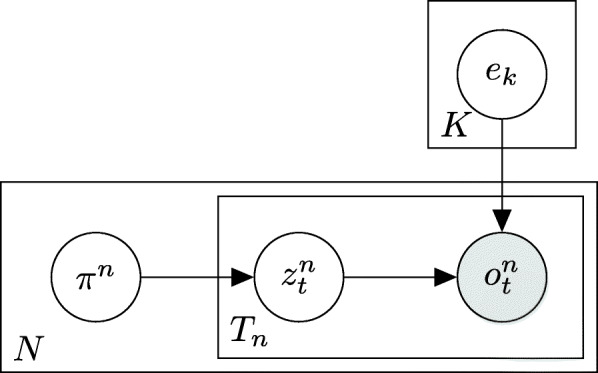
A plate diagram for MMM. The mutations $o_{t}^{n}$ (1≤*t*≤*T*_*n*_;1≤*n*≤*N*) in each sample are observed (gray circles) and are modeled as depending on a latent (empty circles) signature *e*_*k*_. The (latent) selected signature $z_{t}^{n}$ that generated the observed mutation depends on an exposure vector *π*^*n*^ that is selected once for each of the *N* samples

The basic multinomial mixture model we use was presented in [22, 25] and is depicted in Fig. 1. The model is parameterized by the signatures *S*_1_,…,*S*_*K*_ and their *relative exposure* vector *π*, where *π*_*i*_ is the prior probability for the *i*th signature to emit any given mutation.

In the following exposition, we assume for simplicity a single sample to facilitate the generalization to our suggested model presented in the next section. Given the observed mutations *O* and the unobserved signatures *Z*, the model’s likelihood is:

$${}\Pr[\!O] \,=\, \prod\limits_{t=1}^{T} \Pr\left[o_{t}\right] \,=\, \prod\limits_{t=1}^{T} \sum\limits_{i=1}^{K} \Pr\left[o_{t}, z_{t}=i\right] = \prod\limits_{t=1}^{T} \sum\limits_{i=1}^{K} \pi_{i} e_{i}(o_{t}) $$

Denoting by *V*_*j*_=|{*t*|*o*_*t*_=*j*}| the number of times the *j*th category appears in the data, the likelihood can be rewritten as:

$$f(V,\pi,e):=\prod\limits_{j=1}^{M} \left(\sum\limits_{i=1}^{K} \pi_{i} e_{i}(j)\right)^{V_{j}} $$

The likelihood can be maximized using the expectation maximization (EM) algorithm. In the E-step, we compute the expectation of the model’s emissions and (relative) exposures under the current assignment to those parameters. Specifically:

- The expected number of times that signature *i* emitted mutation category *j* is computed by $E_{i}(j, V, \pi, e) := \frac {V_{j} \pi _{i} e_{i}(j)}{\sum _{k=1}^{K} \pi _{k} e_{k}(j)}$.
- Similarly, the expected number of times signature *i* was used is computed by $A_{i}(V, \pi, e) := \sum \limits _{j=1}^{M} E_{i}(j, V, \pi, e)$.

These expectations are normalized (to probabilities) in the M-step to yield a new set of parameters until convergence.

One obvious weakness of this model is that given a collection of samples, we cannot expect all of them to have the same exposures *π*. While it is possible to learn a unique exposure vector per sample, as done by existing methods, the number of parameters then grows linearly with the number of samples, which may lead to overfitting in a sparse data scenario.

### Mix: a mixture of MMMs

**Fig. 2:**
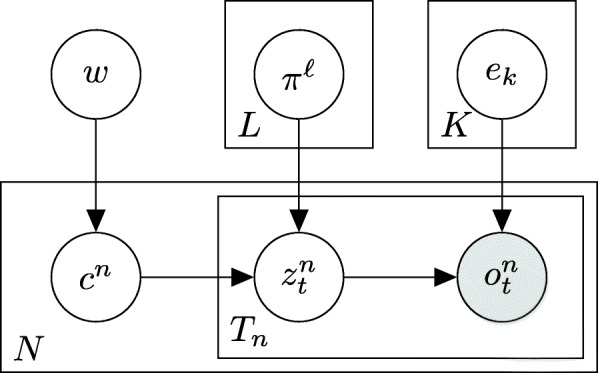
A plate diagram for Mix. Notation is the same as in Fig. 1. The key difference from MMM as illustrated in the plate diagram is that the (latent) selected signature $z_{t}^{n}$ depends on a cluster *c*^*n*^ chosen for each of the samples (with prior probabilities *w*), as well as (latent) cluster exposures *π*^*l*^ shared across samples within that cluster

In order to cope with the problem of sparse data, our approach is to cluster the samples and learn exposures per cluster rather than per sample. To this end, we propose a mixture model and a scheme to optimize its likelihood, leading to simultaneous optimization of sample (soft) clustering, exposures and signatures (Fig. 2). Given a hyper-parameter *L* indicating the number of clusters, denote by $c^{n}\in \{1\dots L\}$ the hidden variables representing the true cluster identity of each sample. Our goal is to learn cluster prior probabilities $w=(w_{1}\dots w_{L})$, cluster exposures $\pi =\left (\pi ^{1}\dots \pi ^{L}\right)$, and shared signatures *e*, so as to maximize the model’s likelihood:

$$\begin{array}{*{20}l} \Pr\left[V|w,\pi,e\right]&=\prod\limits_{n=1}^{N} \Pr\left[V^{n}|w, \pi, e\right]\\ &= \prod\limits_{n=1}^{N} \sum\limits_{\ell=1}^{L}\Pr\left[c^{n}=\ell, V^{n}|w, \pi, e\right]\\ & = \prod\limits_{n=1}^{N} \sum\limits_{\ell=1}^{L} \Pr\left[c^{n}=\ell\right]\Pr\left[V^{n}|\pi^{\ell}, e\right]\\ & = \prod\limits_{n=1}^{N} \sum\limits_{\ell=1}^{L} w_{\ell} f(V^{n}, \pi^{\ell}, e) \end{array} $$

We can generalize the EM algorithm of the previous MMM model to Mix as follows:

- *E-step*: Compute for every *i*,*j*,*n*,*ℓ*:
  - $f^{n,\ell } = \Pr \left [c^{n}=\ell | V^{n}, w, \pi, e\right ] = \frac {w_{\ell }f(V^{n}, \pi ^{\ell }, e)}{\sum \limits _{\ell '=1}^{L} w_{\ell '}f(V^{n}, \pi ^{\ell '}, e)}$
  - $E_{i}(j) = \sum \limits _{n=1}^{N} \sum \limits _{\ell =1}^{L} f^{n,\ell } E_{i}(j, V^{n}, \pi ^{\ell }, e)$
  - $A_{i}^{\ell } = \sum \limits _{n=1}^{N} f^{n,\ell } A_{i}(V^{n}, \pi ^{\ell }, e)$
  - $W_{\ell } = \sum \limits _{n=1}^{N} f^{n,\ell }$
- *M-step*: Compute for every *i*,*j*,*ℓ*:
  - $\pi ^{\ell }_{i} = \frac {A^{\ell }_{i}}{\sum \limits _{i'=1}^{K} A^{\ell }_{i'}}$
  - $e_{i}(j) = \frac {E_{i}(j)}{\sum \limits _{j'=1}^{M} E_{i}(j')}$
  - $w_{\ell } = \frac {W_{\ell }}{\sum \limits _{\ell '=1}^{L} W_{\ell '}}$

A detailed derivation of the algorithm is given in Additional file 1. To learn the model in a refitting setting, i.e., with fixed known signatures, the update step of *e* is skipped and the initial value for it is set to the given signatures. Each EM iteration can be completed in ${\mathcal {O}(NLK)}$ time for *N* samples, *L* clusters and *K* signatures. The EM algorithm is run until it converges to a local maximum and up to 1000 iterations. To avoid being trapped in poor local maxima, we train the model ten times with different random seeds and choose the one that yields the highest likelihood.

To estimate the hyper parameters of Mix (*L*,*K*), we use the Bayesian information criterion (BIC) to weigh the tradeoff between model fit and the number of parameters. We train the model on a range of choices for *L* and *K*, and choose the hyper parameters to be:

$${}L^{*},K^{*} = \underset{L,K}{\text{arg\,min}} \{\texttt{Mix}.size \cdot\log(n) - 2\log(\texttt{Mix}.prob)\} $$

where Mix.*size* is the number of parameters in the model, *n* is the number of data points (number of mutations), and Mix.*prob* is the probability of the data given the trained model. The total number of learned parameters in Mix is given by (*L*−1)+*L*(*K*−1)+*K*(*M*−1), where *M* is the number of mutation categories.

Given a trained model [*w*,*π*,*e*] and a sample *V* we would like to construct an exposure vector *E* for it. We explore two inference schemes:

- *Hard clustering*: we define the exposures based on the most likely cluster for that sample, i.e., *E*=*π*^*ℓ*^ where *ℓ* is the cluster that maximizes *f*^*ℓ*^:= Pr[*c*^*n*^=*ℓ*|*V*,*w*,*π*,*e*].
- *Soft clustering*: we take a weighted sum of all clusters’ exposures, with *f*^*ℓ*^ as weights. Precisely, $E=\sum \limits _{\ell =1}^{L} f^{\ell }\pi ^{\ell }$.

In both cases, *E* is the normalized exposure, i.e summed to 1, so we will multiply it by the number of mutations to obtain the real exposures, although for some applications the normalized exposures performed slightly better. Note that we mostly use hard-clustering to cluster the samples and soft clustering to get the exposures.

We present below both de-novo experiments, in which we learn mutational signatures, as well as *refitting* experiments, in which we assume the signatures are given. In the latter cases, we restrict our analyses to Single Base Substitution (SBS) mutational signatures in COSMIC [26] (https://cancer.sanger.ac.uk/cosmic/signatures_v2.tt) that are known to be active in the cancer type being analyzed.

### Mutation and clinical data

We applied Mix to analyze mutational signatures in three datasets.

#### Somatic mutation data

1. *MSK-IMPACT* [27, 28] *Pan-Cancer.* We downloaded mutations for a cohort of patients with Memorial Sloan Kettering Integrated Mutation Profiling of Actionable Cancer Targets (MSK-IMPACT) targeted sequencing data from https://www.cbioportal.org/. The MSK-IMPACT dataset contains 11,369 pan-cancer patients’ sequencing samples across 410 target genes. We restrict our analysis to the 18 cancer types with more than 100 samples, which results in a dataset of 5931 samples and an average of 6.8 mutations per sample. According to COSMIC [26] there are 17 mutational signatures that are active in those cancer types, 12 of which are associated with more than 5% of the mutations. The 17 active COSMIC signatures are Signatures 1-8, Signatures 10-13, Signatures 15-17, Signature 20, and Signature 21.
2. *ICGC breast cancers (BRCA).* We downloaded mutations for 560 breast cancer patients [29] with whole-genome sequencing data from the International Cancer Genome Consortium. There are about 6214 mutations per sample in this collection and 12 active COSMIC [26] signatures are associated with it. The 12 active COSMIC signatures in breast cancer are 1, 2, 3, 5, 6, 8, 13, 17, 18, 20, 26, and 30.
3. *TCGA ovarian cancers (OV).* We downloaded mutations from whole-exome-sequencing data of 411 ovarian cancer patients from The Cancer Genome Atlas [30]. There are about 113 mutations per sample in this collection and 3 active signatures are associated with it. The 3 active COSMIC [26] signatures are 1, 3, and 5.

In addition, we analyzed mutation data sets for which we had clinical information on homologous recombination deficiency (HRD) status or immunotherapy response:

#### Clinically-oriented data

1. *Whole Genome Sequencing of Triple Negative breast cancers*. Triple negative whole genome breast cancers data along with their HRDetect-predicted labels from Staaf et al. [31]. The output labels are categorized by the probability of HRD: high (HRD score above 0.7), intermediate (0.2 to 0.7), and low (below 0.2). Overall, 139 patients are predicted as “high,” while 13 and 85 are predicted as “intermediate” and “low,” respectively. To make the labels binary, we removed the 13 “intermediate” labeled samples, leaving 224 samples, 62% of them with HRD.
2. *MSK-IMPACT sequencing of Non-small cell lung cancer (NSCLC) data treated by CTLA-4/PD-L1* [32]. We downloaded the data from the cBioPortal [33, 34]. There are 240 NSCLC patients in this cohort. 206 patients went through PD-L1 monotherapy, and 34 patients went through a combined therapy of PD-L1 and CTLA-4. To have a clean dataset to analyze, we used the 150 LUAD patients that were treated with PD-L1 monotherapy and either showed durable clinical benefit (41 samples) or not (109 samples).
3. *MSK-IMPACT sequencing of pan-cancer patients data treated by CTLA4, PD-1, and/or PD-L1* [35]. Last, we downloaded from the cBioPortal [33, 34] 1583 mutation profiles of pan-cancer patients with survival information. In detail, there are 339 non-small cell lung cancers, 311 melanoma, 209 bladder cancers, 137 renal cell carcinoma, 126 head and neck cancers, 115 esophagogastric cancers, 114 gliomas, 109 colorectal cancers, 83 cancers of unknown primary, 39 breast cancers, and 1 skin cancer (non-melanoma). One thousand two hundred forty-three of the patients were treated with PD-1/PD-L1, 95 were treated with CTLA4, and 245 were treated with Combo.

#### Synthetic data simulation

We simulated data according to our model as follows. We start by learning Mix on MSK-IMPACT panel data to obtain realistic estimates for the model’s hyperparameters (10 clusters and 6 signatures using BIC) and parameters (cluster probabilities *w*, signature exposures *π* per cluster and the signatures themselves *e*). We use these estimates as a baseline for data simulation. In the simulations, we vary the number of clusters *L* from 5 to 9, by sampling clusters without replacement using the distribution *w*. We then assign the clusters their corresponding weights from *w*, normalizing the sum to 1 $w=(w_{1},\dots,w_{L})$. Let $\pi =(\pi ^{1},\dots,\pi ^{L})$ denote the learned signature exposures over the selected clusters. Let $p_{k}=\sum _{\ell =1}^{L} w_{\ell }\pi _{k}^{\ell }$. Next, we sample without replacement *K*=4 signatures with probabilities $p_{1},\dots,p_{6}$. Finally, we normalize per cluster the exposures over the selected signatures to sum to 1. We applied this simulation setup to generate 5000 samples, similar to the number of samples in the MSK-IMPACT data. For each sample, we first determine its number of mutations by sampling uniformly (with replacement) a sample from the MSK-IMPACT data and adopting its number of mutations. Last, we use the generative process of Mix to sample mutations.

### Performance evaluation in a refitting scenario

In order to evaluate Mix and other algorithms on their ability to infer accurate mutational signature exposures on sparse data, we focus on whole-genome or whole-exome data where we have information about active signatures. The evaluation procedures require generating sparse, downsampled datasets to imitate the target sequencing data. In this section, we first describe the downsampling procedure, and then how to use it for evaluation.

#### Downsampling strategies

For evaluation purposes, we wished to simulate targeted sequencing panels from higher coverage datasets. We use two downsampling strategies: (i) downsampling WGS/WXS data by constraining the samples to target regions of MSK-IMPACT [27, 28] and (ii) random sampling of an average of *d* mutations per patient. In detail, for each patient *i*, we sample *n*_*i*_∼*Pois*(*d*) and then randomly sample *n*_*i*_ mutations from the mutation set *O*^*i*^ without replacement.

#### Reconstruction error

To compare methods in their ability to learn mutational signature exposures on sparse datasets, we compare the reconstruction error (RE) obtained by each method on a *full* dataset using relative exposures inferred on a *downsampled* dataset. For ease of comparison, we fix the signature matrix *S* to consist of known signatures from COSMIC [26]. Since the full and downsampled datasets have different numbers of mutations, we compare them only on their relative exposures. Let *V* be an *N*×*M* matrix where *V*_*ij*_ is the number of times mutation category *j* is observed in tumor *i* in the full dataset, and let *V* ~ be the matrix *V* normalized so that each row sums to one. Given the *N*×*K* relative exposure matrix *E*_*d*_ computed on the downsampled data, we define the reconstruction error as *RE*:=|*V* ~−*E*_*d*_·*S*|_1_, where |·|_1_ is the L1 norm.

#### Exposure reconstruction error

Another reconstruction error measure we use to compare signature learning from sparse data is exposure reconstruction error (ERE). Using the mutation matrix *V* and the signature matrix in the cancer type *S*, we learn the “true” (i.e., non-relative) exposures *E* using NNLS, which is a common method used to learn exposures from rich data. Again, let *E*_*d*_ be the relative exposures computed on the downsampled data, and let *E* ~ be the *E* normalized so that each row sums to one, we define *ERE*:=|*E* ~−*E*_*d*_|_1_. This measure is better suited to the case were we would like to know the exposures rather than the mutations, as the mutations can be noisy and we do not expect mutational signatures to be able to reconstruct them with no error.

### Implementation details

Mix is implemented in Python 3 using numpy [36]. For NMF and KMeans, we used the scikit-learn implementation [37]. NNLS was taken from scipy [38]. The workflow is managed by Snakemake [39]. The code is available at https://github.com/itaysason/Mix-MMM [40].

## Results

We developed the Mix algorithm for elucidating the mutational signature landscape of input samples from their (sparse) targeted sequencing data. We tested our algorithm on synthetic data, downsampled whole-genome/whole-exome data, and gene-panel data and compared its performance to existing approaches. First, we applied Mix to learn parameters from synthetic data it generated. Second, we used Mix to reconstruct mutational signature exposures from downsampled ICGC breast cancer [29] and TCGA ovarian cancer data [30], also applying it to another downsamled data to cluster samples and predicting homologous recombination deficiency (HRD) status. Third, we applied Mix to the MSK-IMPACT Pan-Cancer targeted sequencing data [27, 28]. We tested its success in discovering mutational signatures and in clustering patients. Finally, we tested Mix in a clinical setting, aiming to predict the benefit of PARP inhibitor therapy for breast cancer patients and the benefit of immunotherapy for lung cancer patients.

### Mix design

In the field of mutational signatures, NMF-based methods like SigProfiler [4] are used, as are statistical analogs of NMF like EMu [14] or signeR [16]. For these methods, the number of parameters grows linearly with the number of patients, as a consequence of learning an exposure vector for each patient. Commonly, when using whole-genome/whole-exome data, each patient has many mutations, spanning most categories of mutations (usually 96 categories), allowing the accurate estimation of these exposures. In the increasingly available case of gene panel data, patients usually have less than 10 mutations, causing most categories to have zero counts leading to a number of parameters that is larger than the number of data points. One method, SigMA [24], which was designed to predict HRD status in breast cancer samples, addresses this challenge by first learning patient clusters on rich data from whole genome sequencing, then associating sparse samples with these clusters using a likelihood score, and finally applying a classifier (that uses the likelihood score along with other features) to predict HRD status.

To solve the sparsity problem, we developed the Mix model. Mix is a probabilistic model that simultaneously learns signatures and soft clusters patients, learning exposures per cluster instead of per sample. Then, to obtain a unique exposure for each new patient, Mix soft-clusters the patient’s mutations and takes a linear combination of all exposures according to their probability. With this, Mix also solves another problem of existing methods, where adding a new patient requires learning a new exposure vector for it. Mix is trained using Expectation Maximization and selects hyperparameters using the Bayesian information criterion. A formal description of Mix, model evaluation strategies and information about the datasets used are given in the “Methods” section.

### Performance on synthetic data

As a first test case of our model, we applied it to synthetic data created to have similar characteristics as the MSK-IMPACT dataset [27, 28]. We evaluated Mix in both estimating the number of clusters and signatures that underlie the data and learning the model’s parameters. The results are summarized in Additional file 1: Table S1 and show that Mix can accurately reconstruct the simulation parameters from sparse data. In 4 out of 5 settings, BIC was a good estimator for the hyperparameters, estimating the exact number of clusters and signatures. In 3 out of these 4 settings, Mix perfectly reconstructed all clusters’ exposures and signatures (average similarity ≥0.97), and in one of these settings, Mix reconstructed 8 out of 9 clusters, and the remaining one was a duplicate. In one setting, Mix underestimated the hyper parameters, learning 5 clusters instead of 8 and 3 signatures out of 4. However, on closer inspection, the missing signature is involved in less than 5% of the mutations. Without this signature there are only 5 clusters with distinct exposures (similarity <0.95), supporting the inferred model.

### Reconstructing mutation and exposure profiles from simulated data

To evaluate Mix in a more realistic, yet controlled setting, we applied it to simulated, downsampled data that is derived from whole-genome or whole-exome sequencing (see the “Methods” section). In this application, the full mutation profiles are available to us and can be used to guide the evaluation.

We focus the experiments on exposure learning (refitting scenario) and fix the signatures to be the known active COSMIC [26] signatures in the given cancer type. We train Mix using downsampled data from 50% of samples, compute exposures on downsampled data for the remaining 50% of (test) samples, and report the average reconstruction error (RE) and exposure reconstruction error (ERE) on the whole mutation catalog of the test samples. We repeat these experiments for average number of mutations per sample *d* ranging from 3 to 18, and the MSK-IMPACT region (panel) mutations, with an average of 5.6 and 4.5 mutations for WGS BRCA [29] downsampling and WXS OV [30] downsampling data, respectively.

**Fig. 3:**
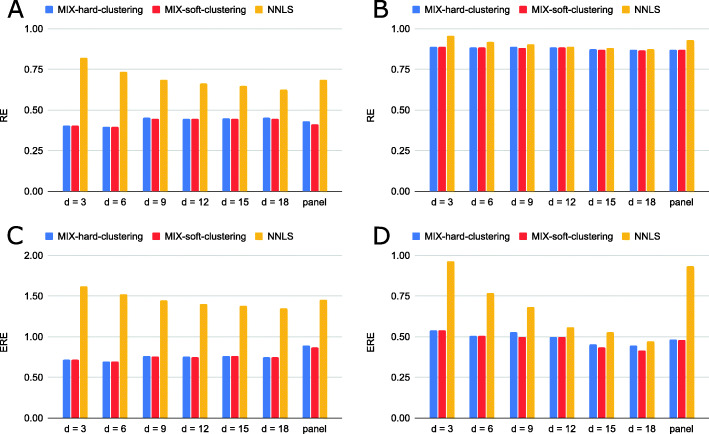
Performance evaluation on simulated data. Shown are reconstruction errors (RE) and exposure reconstruction error (ERE) for Mix (two variants) and NNLS across two datasets, breast cancer (**A**, **C**) and ovarian cancer (**B**, **D**), and seven downsampling schemes

We compare the performance of Mix against the widely-used non-negative least squares (NNLS) approach. Given a mutation count matrix *V* and signatures *H*, NNLS extracts (non-negative) exposures *W* that minimize ∥*V*−*WH*∥_2_. The results are shown in Fig. 3. We include in the comparison also a hard clustering inference scheme for Mix (see the “Methods” section). Out of the two Mix variants, the soft clustering inference of exposures displays better performance, and both outper- form NNLS in all cases. An interesting observation is that we do not see a decrease in reconstruction error when the number of mutations increases. This might be caused by inherent noise in mutation data, which is mitigated when reducing the dimension of the data from mutation categories to signatures.

Although they were not developed to tackle such task, we also compared to three current refitting tools—deconstructSigs [18], SigLasso [41], and YAPSA [42]. SigLasso did not halt on the MSK-IMPACT data, and DeconstructSigs and YAPSA both gave similar results to the NNLS approach. Full comparison results are given in Additional file 1: Tables S2 and S3.

### Comparison to SigMA on clinically-relevant data

Next, we wished to compare between Mix and SigMA, the only previous method for analyzing panel data. To this end, we trained Mix using a panel downsampling version of the BRCA data [29], with the 12 COSMIC [26] signatures that are known to be active in this cancer type. In this application, BIC yields an estimate of 3 clusters which was used in the training of Mix. Notably, SigMA was trained on those 560 BRCA samples, along with 170 additional samples [24].

**Fig. 4:**
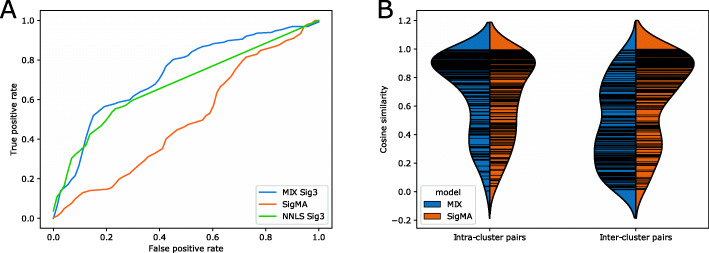
Performance evaluation in a clinical setting. **A** ROC curves for HR deficiency prediction based on Mix, SigMA and NNLS with AUCs of 0.73, 0.5 and 0.68, respectively. **B** Clustering quality of Mix and SigMA as measured by intra-cluster and inter-cluster cosine similarities

We applied both models to panel downsampling of 224 WGS triple negative breast cancer samples [31], clustering them and predicting their HRD status. Signature 3 activity is known to be a good predictor of HRD [43], with 0.96 AUC on this dataset when estimating its exposure using NNLS on the full (WGS) mutation data. For Mix, we based our status estimate on Signature 3 exposure using the soft clustering variant. For SigMA, we used the Signature 3 mva output. For completeness, we also evaluated an NNLS estimate of Signature 3 exposure on the panel downsampling data. The HRD status prediction ROC curves of the three methods are depicted in Fig. 4A, with Mix showing a clear advantage over the two competing methods. When considering the performance of the three methods at low false positive rates (FPRs), we observe 31% true positive rate (TPR) for Mix at 10% FPR, which is on par with NNLS (34%) and higher than SigMA (12%); for an FPR of 20% the TPR of Mix increases to 58%, outperforming NNLS (48%) and SigMA (34%).

Next, we evaluated the clustering produced by Mix and SigMA. To this end, we focused on the clustering produced by the hard clustering variant of Mix and compared to the “categ” output of SigMA. For each method, we randomly drew 200 intra-cluster sample pairs and 200 inter-cluster sample pairs and compared the distributions of similarities they induce. Specifically, for each pair, we computed the cosine similarity between their exposures in the WGS data, obtained by NNLS with the 12 known COSMIC [26] signatures in breast cancer. As can be seen in Fig. 4B, the intra-cluster pairs of Mix displayed substantially higher similarity than inter-cluster pairs (0.69 vs. 0.46), while no such difference was observed for SigMA (0.65 vs. 0.66).

### Learning signatures and patient classes from MSK-IMPACT

**Fig. 5:**
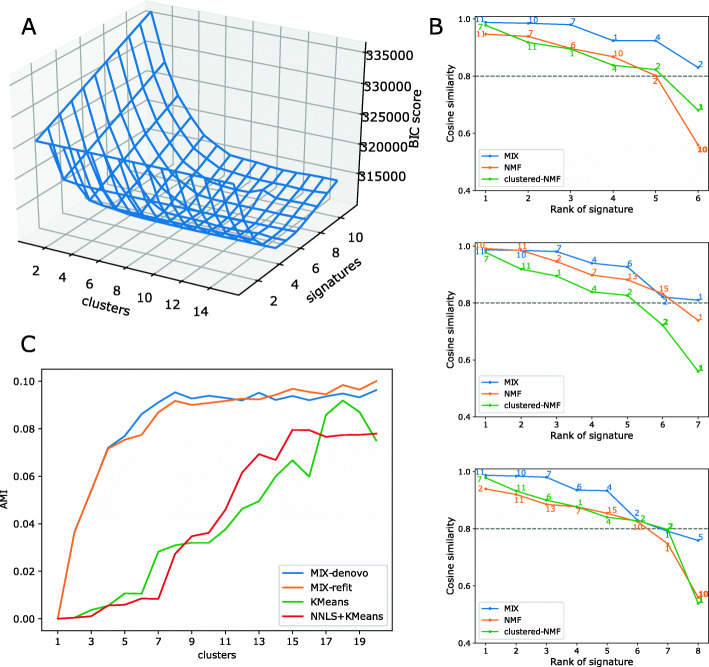
Performance evaluation on MSK-IMPACT data. **A** Hyper-parameter selection in Mix. A plot of BIC score (*y*-axis) as a function of the number of signatures (*z*-axis) and the number of clusters (x-axis). **B** De-novo signature discovery from MSK-IMPACT panel data [27, 28]. Shown are sorted cosine similarities between learned signatures and most similar COSMIC [26] signature (denoted next to the plot) for Mix, NMF, and clustered NMF across a range of number of signatures (6-8 corresponding to top, middle and bottom, respectively). Repeating signatures of the same model are in bold. **C** AMI score as a function of the number of clusters for each model

Moving to real data, we applied Mix to analyze 5931 samples from the MSK-IMPACT dataset [27, 28]. We trained Mix with ten random initializations on number *L* of clusters ranging from 1 to 15 and number *K* of signatures ranging from 1 to 12 (up to 12 signatures are associated with these data according to COSMIC, see the “Methods” section). Using BIC, we found *L*=10 and *K*=6 to be the optimal hyper parameters (Fig. 5A). We also trained a refitting version of Mix on this dataset with the known 17 COSMIC [26] signatures and found *L*=7 using BIC. The learned signatures can be viewed in Additional file 1: Figure S1 and the clusters’ exposure of both de-novo and refitting models can be seen in Additional file 1: Figure S2. We observed that the BIC score is affected mostly by the number of signatures, with a clear minimum between 5 and 7, but less so by the number of clusters.

To evaluate the learned signatures, we compared them to the COSMIC [26] signatures using the cosine similarity measure (Fig. 5B and Additional file 1: Figure S3). Mix accurately reconstructed 6-8 known signatures with cosine similarity >0.8 as commonly required [15]. We compared our performance to that of the standard NMF algorithm as well as to a clustered variant where we first form meta-samples corresponding to each of the 18 cancer types, and then apply the NMF to these meta-samples. To form a meta-sample, we combined together all mutations of samples that belong to the corresponding cancer types (i.e., sum together the samples’ mutation counts). For these additional applications, we varied the number of signatures from 6 to 8; for Mix we optimized the number of clusters in each application using BIC as described above. Further, we executed each algorithm ten times with different (random) initializations and chose the run that yielded the best score (likelihood for Mix or approximation error for NMF). Evidently, Mix dominates the other approaches across the explored range, yielding a larger number of highly accurate and distinct signatures. Notably, Mix was consistently able to identify the following signatures: Aging (Signature 1), APOBEC (Signatures 2), Smoking (Signature 4), MMR (Signatures 6), UV (Signature 7), POLE (Signature 10), and TMZ (Signature 11). All these signatures are supported by a previous refitting analysis that was based on the known COSMIC signatures [28].

We note that we also tried comparing Mix to SigProfiler [4], SigAnalyzer [44, 45], SomaticSignatures [46], and MutationalPatterns [19], which are tools for learning mutational signatures. We executed all these tools using their default settings. SigProfiler and MutationalPatterns were too time consuming (expected running time of days to weeks to perform the experiments described here). SignatureAnalyzer gave inferior results to the NMF application reported here (only two signatures, 1 and 7, consistently recovered with cosine similarity greater than 0.8). Similarly, SomaticSignatures performed consistently worse than the NMF implementation reported here (Additional file 1: Figure S4). Expectedly, Mix outperformed the other tools that were not developed to handle sparse data

Next, we used Mix to cluster samples, choosing for each sample the cluster with maximal posterior probability. We scored the resulting clusters against a benchmark clustering of the samples according to their cancer type with the adjusted mutual information (AMI) score (Fig. 5C). We note that in addition to validating our results, predicting cancer type from targeted sequencing panels has potential clinical relevance, as approximately 3% of tumors are of unknown primary origin [47] and there has been a recent focus on developing methods to predict cancer type using mutations [48, 49].

We compared our results to those obtained by KMeans clustering of the original mutation count vectors as well as to a refined variant where we first apply NNLS to the data using the 17 active COSMIC [26] signatures, then cluster the resulting exposures using KMeans. For Mix we present an additional refitting variant where we set the signatures to be the 17 COSMIC signatures. For all methods, we report results with *L*=1−20 clusters; for de-novo Mix we choose the number of signatures for each value of *L* using BIC. As the clustering of specific samples depends on their sparsity, we also report AMI scores when focusing on samples with at least 10 mutations. Figure 5C demonstrates that the two Mix variants outperform the alternative methods in both settings. Interestingly, the two Mix variants display similar performances, suggesting that Mix can cluster well even without prior knowledge. The fact that the Mix AMI scores seem to converge for 6 clusters or more suggests that Mix is robust to the number of clusters being used.

### Predicting immunotherapy response of lung cancer patients

As another challenge, we wished to test the utility of Mix in additional clinical scenarios in which signature analysis is less abundant. Specifically, we applied Mix to 150 LUAD samples [32] to predict durable clinical benefit to PD-L1 monotherapy treatment. We used the same de-novo and refitting Mix models that were trained on the MSK-IMPACT pan-cancer data [27, 28] in previous section. Notably, most samples in the MSK-IMPACT data set are from LUAD patients (1277, 21%).

Tumor mutational burden (TMB) is one of the most widely known and analyzed genomic correlates with immunotherapy response. In addition, Rizvi et al. [50] found that the exposure of Signature 4 was associated with response in non-small cell lung cancer. For the former, as we have targeted sequencing data, we used the plain mutation counts instead which were shown to be in high correlation with TMB [51]. For the latter, we used Mix to obtain signature exposures and compared to those derived using NNLS on the 17 COSMIC [26] signatures active in the MSK-IMPACT dataset [27, 28].

We evaluated the performance of each method using the area under the ROC curve (AUC). Specifically, we report the AUC score of Signature 4 at predicting the treatment response. For Mix in the de-novo setting, we report the signature which is most similar to signature 4, with cosine similarity 0.924. The AUC scores are 0.64, 0.63, 0.63, 0.6 for refitting-Mix, de-novo-Mix, NNLS, and TMB, respectively. The results suggest a slight advantage for Mix over alternative approaches in this setting.

## Discussion

An important application of Mix is for predicting the potential benefit of drug treatments based on the inferred activities of relevant mutational signatures. In particular, we have shown the utility of our model for predicting HRD status and hence the benefit of treatment with PARP inhibitors. However, our results were on a downsampled whole-genome sequencing dataset where “ground truth” was determined by the HRDetect algorithm [11]. While HRDetect has shown promise at predicting response to PARP inhibitors [11] and in stratifying triple-negative breast cancers by outcome [31], there may be value in training Mix on other HRD classifications or investigating discrepancies between Mix and HRDetect. Ultimately, the clinical value of Mix will be better determined when it can be evaluated on a dataset with sequencing data and PARP response. We also showed the utility of our model for predicting the response to immunotherapy. To our knowledge, this is the first time targeted sequencing data was used in this setting, yielding promising results that merit further research.

Beyond the prediction of signature exposures, Mix has the advantage of clustering the patients to potentially clinically relevant groups. To showcase this relevance, we conducted a survival analysis of 1583 pan-cancer patients from [35] whose mutation profiles are not used for the training process of Mix. We applied de-novo and refitting Mix models that were trained on the MSK-IMPACT pan-cancer data [27, 28] and assigned Mix cluster memberships to patients via hard clustering, i.e., each patient is assigned to the most likely cluster. To pinpoint clinically relevant clusters, we used Cox regression, corrected for cancer type, age, gender, and TMB.

**Fig. 6:**
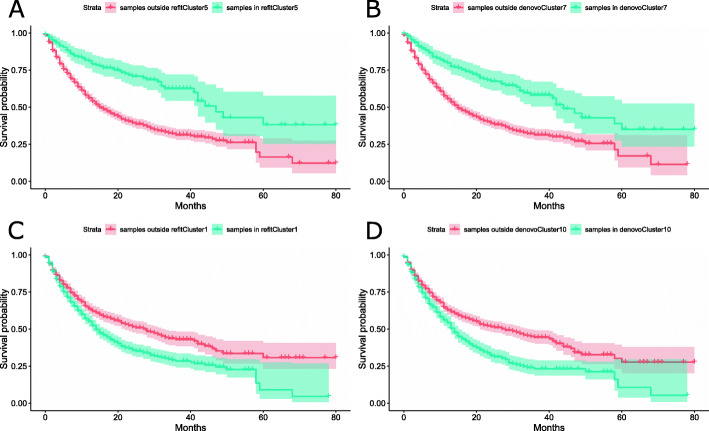
Survival analysis of Mix patient clusters. Kaplan-Meier plots for refitting Mix cluster 5, de-novo Mix cluster 7, refitting Mix cluster 1 and de-novo Mix cluster 10 corresponding to **A**–**D** respectively

Out of the seven refitting clusters, we find that patients in cluster 5 have significantly better survival (Fig. 6A), with *p* value of 0.027. Our analysis indicates that the dominant signature in this cluster with exposure of 0.82 is Signature 7, which is associated with UV-radiation. At the same time, 131/204 patients in the cluster are Cutaneous Melanoma (SKCM) patients, which agrees well with the previous finding that Signature 7 is correlated with better SKCM survival [52]. Out of the remaining 73 samples in the cluster, 40 are Melanoma of another sub-type and 8 are of unknown primary origin (UPO). Reassuringly, we find a corresponding cluster also in the de-novo setting, where out of ten clusters, patients in cluster 7 have significantly better survival, with *p* value of 0.03. This cluster is again dominated by Signature 7 (exposure of 0.9) and 149/243 of its members are SKCM patients (Fig. 6B). Similarly to the refitting case, in this cluster, there are 44 other Melanoma samples and 8 UPO samples.

In addition, we find that patients in refitting cluster 1 and de-novo cluster 10 have significantly worse survival (Fig. 6C, D), with *p* values of 0.008 and 0.004, respectively. These clusters contain 754 (48%) and 617 (39%) of the samples, respectively, spanning all cancer types. They are characterized by samples with the lowest average patient TMB (among the different clusters) with a median value of 4, as is expected for patients treated with immunotherapy [53] given the relationship between tumor mutation burden and neoantigen load.

While we have shown promising results for Mix, one limitation of our model is that the number of clusters should be larger than the number of signatures or else a possible solution would be to assign each cluster only one signature, which will serve as the “average” signature across samples in that cluster. This property could in fact become an advantage if we only wish to cluster samples, in which case we could set the number of clusters to be equal to the number of signatures and require a single signature with an exposure of 1 in each cluster.

## Conclusions

Sparse mutation data, as characteristic of targeted sequencing assays, is becoming increasingly available in the clinical setting with important applications in diagnosis and therapy. In this paper, we have presented a novel algorithm to model such data and derive the underlying mutational signatures, exposures, and clinically relevant predictions. Our model is the first to directly capture sparse data without the need for pre-training on rich datasets. We have shown its utility in a range of tasks as well as its favorable performance in comparison to existing methods.

Importantly, we have shown the clinical relevance of our model for predicting HRD status in breast cancer, predicting immunotherapy response in lung cancer and patient stratification. Nevertheless, our model is only a first step in such an analysis that should be followed by specific predictors for the tasks at hand that can take additional data (beyond signature exposure) into account. Our model can be further strengthened by making use of WGS/WXS data, when such are available, to improve the signature discovery step.

## Supplementary Information

**Additional file 1** **Supplementary methods. Table S1** Model reconstruction from synthetic data. **Table S2:** Reconstruction Error (RE) comparison in BRCA and OV. **Table S3:** Exposure Reconstruction Error (ERE) comparison in BRCA and OV. **Figure S1:** De-novo signatures from MSK-IMPACT. **Figure S2:** Clusters learned from MSK-IMPACT. **Figure S3:** Signature discovery from MSK-IMPACT. **Figure S4:** Signature discovery comparison with SomaticSignatures.

## Data Availability

The code is available at https://github.com/itaysason/Mix-MMM [40].
