# Supplementary material for: A mixture model for signature discovery from sparse mutation data
Source: Genome Med. 2021 Nov 1;13:173. doi: 10.1186/s13073-021-00988-7 (PMC8559697; doi:10.1186/s13073-021-00988-7)
Supplement: Supplementary file 1 — Additional file 1 Supplementary methods. Table S1 Model reconstruction from synthetic data. Table S2: Reconstruction Error (RE) comparison in BRCA and OV. Table S3: Exposure Reconstruction Error (ERE) comparison in BRCA and OV. Figure S1: De-novo signatures from MSK-IMPACT. Figure S2: Clusters learned from MSK-IMPACT. Figure S3: Signature discovery from MSK-IMPACT. Figure S4: Signature discovery comparison with SomaticSignatures. [file 13073_2021_988_MOESM1_ESM.pdf]

A mixture model for signature discovery from sparse mutation data

Sason et al.

Supplementary Information

Derivation of the `Mix` EM algorithm

The `Mix` model is parameterized by $\theta = (w, \pi, e)$, where w denotes the cluster probabilities, π is a collection of signature exposures for each cluster, and e represents the signatures that are shared among all clusters. For convenience, we denote by $\theta^\ell = (\pi^\ell, e)$ the parameters that are relevant for cluster ℓ. The model's hyper-parameters are L, K, which denote the number of clusters and the number of signatures. In addition, we denote by N the number of samples and by M the number of mutation categories. In the derivation below, n, ℓ, k, m are the indices that run on $[N], [L], [K], [M]$ respectively. Finally, we denote the observed mutation data as $O = O^1 \dots O^N$ where $O^i = o^i_1 \dots o^i_{T_i}$ are the mutations of sample i; and the hidden cluster and signature identity data as $H = C, Z$ where $C = c^1 \dots c^N$ are the clusters of each of the samples and $Z = Z^1 \dots Z^N$, $Z^i = z^i_1 \dots z^i_N$ are the signatures that underlie each mutation in each sample. For convenience, we omit indices when possible and denote by w, z, o general variables for cluster, signature and mutation respectively. Formally,

- $\forall \ell \quad w_\ell = \Pr\left[c^n = \ell | \theta\right]$, $\sum_\ell w_\ell = 1$
- $\forall \ell, k \quad \pi^\ell_k = \Pr\left[z^n_t = k | w^n = \ell, \theta\right] = \Pr\left[z = k | \theta^\ell\right]$, $\sum_k \pi^\ell_k = 1$
- $\forall k, m \quad e_k(m) = \Pr\left[o^n_t = m | z^n_t = k, \theta\right]$.

For convenience, for every possible choise of hidden data H we denote:

- $\forall \ell \quad W_\ell(H) = |\{n | w^n = \ell\}|$
- $\forall \ell, k \quad A^\ell_k(H) = |\{(n,t) | w^n = \ell, z^n_t = k\}|$
- $\forall k, m \quad E_k(m, H) = |\{(n,t) | z^n_t = k, o^n_t = m\}|$

The log likelihood is given by:

$$\log \Pr\left[H, O | \theta\right] = \sum_\ell W_\ell(H) \log w_\ell + \sum_\ell \sum_k A^\ell_k(H) \log \pi^\ell_k + \sum_k \sum_m E_k(m, H) \log e_k(m)$$

The Q function to maximize (expected complete log likelihood) is given by:

$$\begin{aligned}
Q(\theta|\theta^0) &= \sum_H \Pr\left[H | O, \theta^0\right] \log \Pr\left[H, O | \theta\right] \\
&= \sum_\ell \sum_H \Pr\left[H | O, \theta^0\right] W_\ell(H) \log w_\ell + \sum_\ell \sum_k \sum_H \Pr\left[H | O, \theta^0\right] A^\ell_k(H) \log \pi^\ell_k \\
&+ \sum_k \sum_m \sum_H \Pr\left[H | O, \theta^0\right] E_k(m, H) \log e_k(m) \\
&= \sum_\ell W_\ell \log w_\ell + \sum_\ell \sum_k A^\ell_k \log \pi^\ell_k + \sum_k \sum_m E_k(m) \log e_k(m)
\end{aligned}$$

Where

$$W_\ell = \sum_H \Pr\left[H | O, \theta^0\right] W_\ell(H) \quad A^\ell_k = \sum_H \Pr\left[H | O, \theta^0\right] A^\ell_k(H) \quad E_k(m) = \sum_H \Pr\left[H | O, \theta^0\right] E_k(m, H)$$

We will now show that the Q function is maximized for the M-step given in the Methods under the following restrictions:

$$\sum_{\ell} w_{\ell} = 1, \quad \forall \ell \ \sum_{k} \pi_{k}^{\ell} = 1, \quad \forall k \ \sum_{m} e_{k}(m) = 1$$

First we will show some useful probabilities:

- $\Pr\left[z=k, o=m|\theta^{\ell}\right] = \Pr\left[z=k|\theta^{\ell}\right]\Pr\left[o=m|z=k,\theta^{\ell}\right] = \pi_{k}^{\ell} e_{k}(m)$
- $\Pr\left[o=m|\theta^{\ell}\right] = \sum_{k}\Pr\left[z=k, o=m|\theta^{\ell}\right] = \sum_{k}\pi_{k}^{\ell} e_{k}(m)$
- $\Pr\left[z=k|o=m,\theta^{\ell}\right] = \frac{\Pr\left[z=k|\theta^{\ell}\right]\Pr\left[o=m|z=k,\theta^{\ell}\right]}{\Pr\left[o=m|\theta^{\ell}\right]} = \frac{\pi_{k}^{\ell} e_{k}(m)}{\sum_{k}\pi_{k'}^{\ell} e_{k'}(m)}$
- $\Pr\left[c=\ell, O|\theta\right] = \Pr\left[c=\ell|\theta\right]\Pr\left[O|\theta^{\ell}\right] = w_{\ell}\prod_{t}\Pr\left[o_t|\theta^{\ell}\right]$
- $\Pr\left[O|\theta\right] = \sum_{\ell}\Pr\left[c=\ell|\theta\right]\Pr\left[O|\theta^{\ell}\right] = \sum_{\ell} w_{\ell}\prod_{t}\Pr\left[o_t|\theta^{\ell}\right]$
- $\Pr\left[c^{\ell}|O,\theta\right] = \frac{\Pr\left[c=\ell|\theta\right]\Pr\left[O|\theta^{\ell}\right]}{\Pr\left[O|\theta\right]} = \frac{w_{\ell}\prod_{t}\Pr\left[o_t|\theta^{\ell}\right]}{\sum_{\ell'} w_{\ell'}\prod_{t}\Pr\left[o_t|\theta^{\ell'}\right]}$

We will now show how to compute the variables, note we will omit the 0 index from θ_0, we use I_* as the indicator of some outcome:

$$\begin{aligned} W_{\ell} &= \sum_{H}\Pr\left[H|O,\theta\right] W_{\ell}(H) = \sum_{n}\sum_{H^n}\Pr\left[H^n|O^n,\theta\right] I_{c^n=\ell} \\ &= \sum_{n}\sum_{Z^n}\Pr\left[c^n=\ell, Z^n|O^n,\theta\right] I_{c^n=\ell} = \sum_{n}\Pr\left[c^n=\ell|O^n,\theta\right] \end{aligned}$$

$$\begin{aligned} A_{k}^{\ell} &= \sum_{H}\Pr\left[H|O,\theta\right] A_{k}^{\ell}(H) = \sum_{n}\sum_{H^n}\Pr\left[H^n|O^n,\theta\right] A_{k}^{\ell}(H) I_{c^n=\ell} \\ &= \sum_{n}\sum_{Z^n}\Pr\left[c_n=\ell, Z^n|O^n,\theta\right] A_{k}^{\ell}(Z^n) = \sum_{n}\Pr\left[c_n=\ell|O^n,\theta\right]\sum_{Z^n}\Pr\left[Z^n|O^n,\theta^{\ell}\right] A_{k}^{\ell}(Z^n) \\ &= \sum_{n}\Pr\left[c_n=\ell|O^n,\theta\right]\sum_{Z^n}\Pr\left[Z^n|O^n,\theta^{\ell}\right]\left(\sum_{t} I_{z_t^n=k}\right) \\ &= \sum_{n}\Pr\left[c_n=\ell|O^n,\theta\right]\sum_{t}\sum_{Z^n}\Pr\left[Z^n|O^n,\theta^{\ell}\right] I_{z_t^n=k} \\ &= \sum_{n}\Pr\left[c_n=\ell|O^n,\theta\right]\sum_{t}\sum_{Z^n|z_t^n=k}\Pr\left[Z^n|O^n,\theta^{\ell}\right] \\ &= \sum_{n}\Pr\left[c_n=\ell|O^n,\theta\right]\sum_{t}\Pr\left[z_t^n=k|O^n,\theta^{\ell}\right] \\ &= \sum_{n}\Pr\left[c_n=\ell|O^n,\theta\right]\sum_{t}\Pr\left[z=k|o=o_t^n,\theta^{\ell}\right] \end{aligned}$$

Lastly:

$$E_k(m) = \sum_H \Pr\left[H|O,\theta\right] E_k(m,H) = \sum_n \sum_{H^n} \Pr\left[H^n|O^n,\theta\right] E_k(m,H^n)$$

$$= \sum_n \sum_\ell \sum_{H^n|c^n=\ell} \Pr\left[H^n|O^n,\theta\right] E_k(m,H^n) = \sum_n \sum_\ell \sum_{Z^n} \Pr\left[c^n=\ell, Z^n|O^n,\theta\right] E_k(m,Z^n)$$

$$= \sum_n \sum_\ell \Pr\left[c_n=\ell|O^n,\theta\right] \sum_{Z^n} \Pr\left[Z^n|O^n,\theta^\ell\right] E_k(m,Z^n)$$

$$= \sum_n \sum_\ell \Pr\left[c_n=\ell|O^n,\theta\right] \sum_{Z^n} \Pr\left[Z^n|O^n,\theta^\ell\right] \left(\sum_t I_{z_t^n=k,o_t^n=m}\right)$$

$$= \sum_n \sum_\ell \Pr\left[c_n=\ell|O^n,\theta\right] \sum_t \sum_{Z^n} \Pr\left[Z^n|O^n,\theta^\ell\right] I_{z_t^n=k,o_t^n=m}$$

$$= \sum_n \sum_\ell \Pr\left[c_n=\ell|O^n,\theta\right] \sum_{t|o_t^n=m} \sum_{Z^n|z_t^n=k} \Pr\left[Z^n|O^n,\theta^\ell\right]$$

$$= \sum_n \sum_\ell \Pr\left[c_n=\ell|O^n,\theta\right] \sum_{t|o_t^n=m} \Pr\left[z=k|o=m,\theta^\ell\right]$$

$$= \sum_n \sum_\ell \Pr\left[c_n=\ell|O^n,\theta\right] |\{t|o_t^n=m\}| \Pr\left[z=k|o=m,\theta^\ell\right]$$

This completes the derivation.

Supplementary Figures and Tables

Table S1: Model reconstruction from synthetic data.

Simulated hyperparameters	BIC inferred hyperparameters	Average cluster similarity	# Unique clusters	Average signature similarity	# Unique signatures
(5, 4)	(5, 4)	0.97	5	0.99	4
(6, 4)	(6, 4)	0.99	6	0.99	4
(7, 4)	(7, 4)	1.00	7	1.00	4
(8, 4)	(5, 3)	1.00	5	0.99	3
(9, 4)	(9, 4)	1.00	8	1.00	4

Table S2: Reconstruction Error (RE) comparison in BRCA and OV.

	MIX-hard-clustering	MIX-soft-clustering	NNLS	DeconstructSigs	YAPSA
BRCA d = 3	0.40	0.40	0.82	0.79	0.82
BRCA d = 6	0.39	0.39	0.73	0.74	0.73
BRCA d = 9	0.45	0.45	0.69	0.70	0.69
BRCA d = 12	0.45	0.45	0.66	0.68	0.66
BRCA d = 15	0.45	0.45	0.65	0.67	0.65
BRCA d = 18	0.45	0.45	0.62	0.65	0.62
BRCA panel	0.43	0.41	0.69	0.69	0.69
OV d = 3	0.89	0.89	0.96	0.96	0.96
OV d = 6	0.89	0.89	0.92	0.92	0.92
OV d = 9	0.89	0.88	0.90	0.90	0.90
OV d = 12	0.89	0.89	0.89	0.89	0.89
OV d = 15	0.88	0.87	0.88	0.88	0.88
OV d = 18	0.87	0.87	0.87	0.87	0.87
OV panel	0.87	0.87	0.93	0.93	0.93

Table S3: Exposure Reconstruction Error (ERE) comparison in BRCA and OV.

	MIX-hard-clustering	MIX-soft-clustering	NNLS	DeconstructSigs	YAPSA
BRCA d = 3	0.72	0.72	1.62	1.66	1.62
BRCA d = 6	0.70	0.70	1.53	1.59	1.53
BRCA d = 9	0.76	0.75	1.45	1.51	1.45
BRCA d = 12	0.75	0.75	1.40	1.47	1.40
BRCA d = 15	0.76	0.76	1.38	1.45	1.38
BRCA d = 18	0.75	0.75	1.35	1.42	1.35
BRCA panel	0.89	0.87	1.45	1.52	1.45
OV d = 3	0.54	0.54	0.97	0.97	0.97
OV d = 6	0.50	0.50	0.77	0.79	0.77
OV d = 9	0.53	0.50	0.68	0.70	0.68
OV d = 12	0.50	0.50	0.56	0.58	0.56
OV d = 15	0.45	0.43	0.53	0.55	0.53
OV d = 18	0.44	0.41	0.47	0.50	0.47
OV panel	0.48	0.48	0.93	0.95	0.93

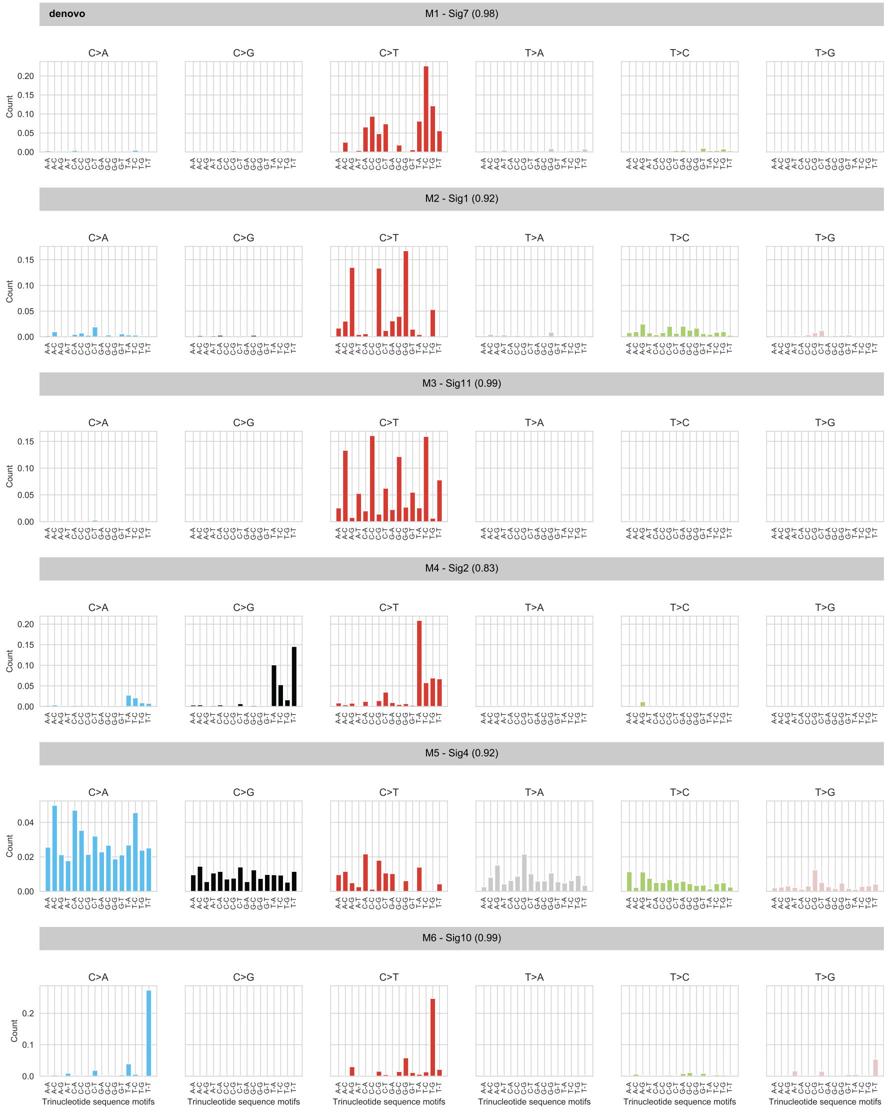

Fig S1: De-novo signatures from MSK-IMPACT. Shown are distributions for the 6 de-novo signatures learned from MSK-IMPACT using `Mix`. For each signature M1-M6 we also indicate the most similar COSMIC signature and the cosine similarity.

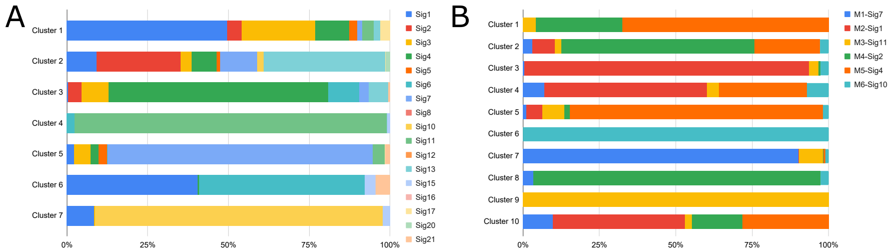

Fig S2: Clusters learned from MSK-IMPACT. Shown are signature distributions in the clusters `Mix` learned from MSK-IMPACT. A) 7 refitting clusters learned using the known 17 active COSMIC signatures. B) 10 clusters learned de-novo.

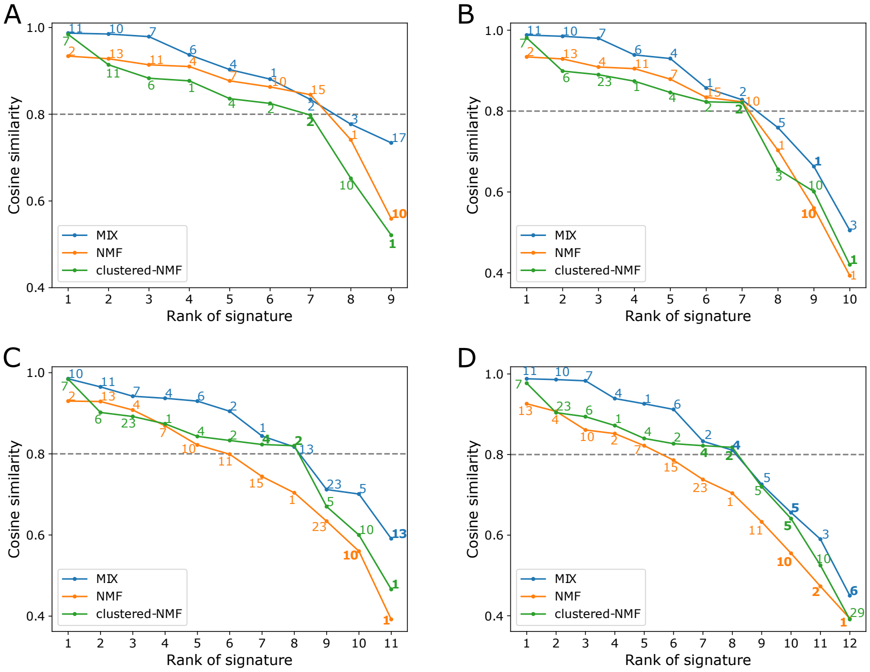

Fig S3: Signature discovery from MSK-IMPACT. Extended results related to Figure 2B. De-novo signature discovery from MSK-IMPACT panel data. Shown are sorted cosine similarities between learned signatures and most similar COSMIC signature (denoted next to the plot) for `Mix`, NMF and clustered NMF across a range of number of signatures (9-12 corresponding to A-D, respectively). Repeating signatures of the same model are in bold.

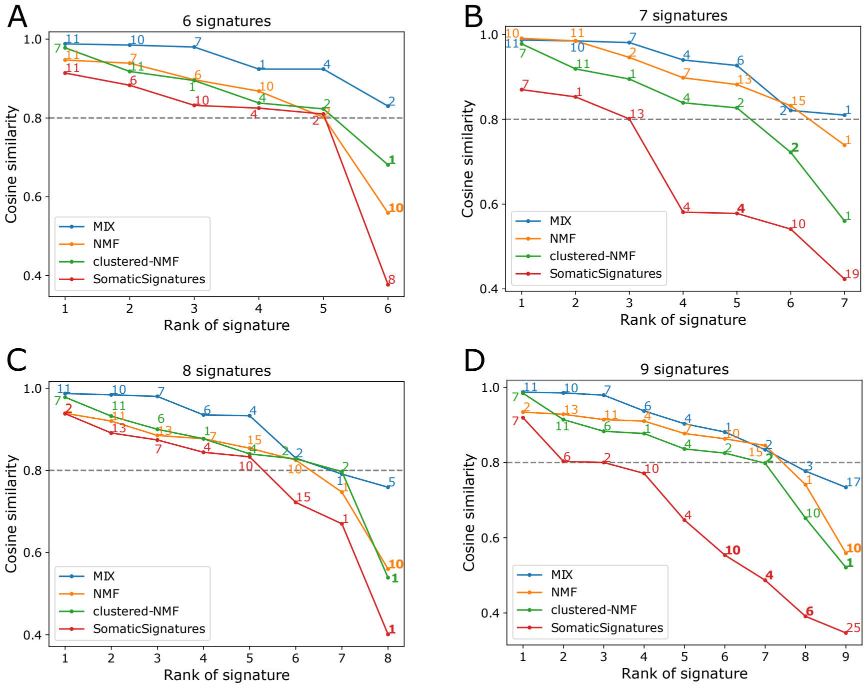

Fig S4: Signature discovery comparison with SomaticSignatures. Extended results related to Figure 2B. De-novo signature discovery from MSK-IMPACT panel data. Shown are sorted cosine similarities between learned signatures and most similar COSMIC signature (denoted next to the plot) for `Mix`, NMF, clustered NMF and SomaticSignatures across a range of number of signatures (6-9 corresponding to A-D, respectively). Repeating signatures of the same model are in bold.
